# Development of a New Binary Matrix for the Comprehensive Analysis of Lipids and Pigments in Micro- and Macroalgae Using MALDI-ToF/ToF Mass Spectrometry

**DOI:** 10.3390/ijms25115919

**Published:** 2024-05-29

**Authors:** Mariachiara Bianco, Giovanni Ventura, Davide Coniglio, Antonio Monopoli, Ilario Losito, Tommaso R. I. Cataldi, Cosima D. Calvano

**Affiliations:** 1 Dipartimento di Chimica, Università degli Studi di Bari Aldo Moro, Campus Universitario, Via E. Orabona, 4, 70126 Bari, Italy; mariachiara.bianco@uniba.it (M.B.); giovanni.ventura@uniba.it (G.V.); davide.coniglio@uniba.it (D.C.); antonio.monopoli@uniba.it (A.M.); ilario.losito@uniba.it (I.L.); tommaso.cataldi@uniba.it (T.R.I.C.); 2 Centro Interdipartimentale di Spettrometria di MAssa per Ricerche Tecnologiche (SMART), Università degli Studi di Bari Aldo Moro, Campus Universitario, Via E. Orabona, 4, 70126 Bari, Italy

**Keywords:** MALDI MS/MS, chlorophylls, lipids, alga, binary matrix

## Abstract

While edible algae might seem low in fat, the lipids they contain are crucial for good health and preventing chronic diseases. This study introduces a binary matrix to analyze all the polar lipids in both macroalgae (Wakame—*Undaria pinnatifida*, Dulse—*Palmaria palmata*, and Nori—*Porphyra* spp.) and microalgae (Spirulina—*Arthrospira platensis*, and Chlorella—*Chlorella vulgaris*) using matrix-assisted laser desorption ionization mass spectrometry (MALDI-MS). The key lies in a new dual matrix made by combining equimolar amounts of 1,5-diaminonaphthalene (DAN) and 9-aminoacridine (9AA). This combination solves the limitations of single matrices: 9AA is suitable for sulfur-containing lipids and acidic phospholipids, while DAN excels as an electron-transfer secondary reaction matrix for intact chlorophylls and their derivatives. By employing the equimolar binary matrix, a wider range of algal lipids, including free fatty acids, phospholipids, glycolipids, pigments, and even rare arsenosugarphospholipids were successfully detected, overcoming drawbacks related to ion suppression from readily ionizable lipids. The resulting mass spectra exhibited a good signal-to-noise ratio at a lower laser fluence and minimized background noise. This improvement stems from the binary matrix’s ability to mitigate in-source decay effects, a phenomenon often encountered for certain matrices. Consequently, the data obtained are more reliable, facilitating a faster and more comprehensive exploration of algal lipidomes using high-throughput MALDI-MS/MS analysis.

## 1. Introduction

Micro and macroalgae are gaining popularity worldwide due to their nutritional and functional properties. This has led to increased consumption, even in Western countries, over the past two decades [1]. Algae are a potential source of high-quality protein and bioactive compounds. They may have numerous health benefits such as antioxidant, antidiabetic, immunomodulatory, antihypertensive, and antihyperlipidemic effects in humans and mammals and could be useful in fighting modern diseases such as obesity and diabetes [2]. The active components are antioxidant scavengers and anti-inflammatory lipid mediators such as carotenoids and polyunsaturated fatty acids (PUFAs) such as eicosapentaenoic acid/docosahexaenoic acid (EPA/DHA); phenolics; fucoidans; numerous pigments such as phycobilins and phycocyanins; and some vitamins such as folate [3,4]. Their integration into the human diet would positively contribute to a more sustainable world because of their roles in carbon dioxide fixation, their rapid growth, and the reduction of land use for agricultural practices since they are suitable for cultivation in waterbodies. Despite the numerous advantages of algae, analyzing their complex lipid composition poses a challenge. Traditionally, solvent extraction methods involving chloroform, methanol, or combinations of these solvents are used [5,6] Thus, in a typical lipidomic study, mass spectrometry (MS) allows for the elucidation of different lipid species [7,8,9]. Several studies on algae lipids employ liquid chromatography (LC) coupled with electrospray ionization (ESI) MS describing major free PUFAs [3,10,11], phospholipids (PLs), glycolipids (GLs) such as digalactosyldiacylglycerols (DGDGs), sulfoquinovosyl diacylglycerols (SQDGs) [12,13], non-polar glycerolipids or minor arsenosugar phospholipids [14,15], or uncommon lipids such as arsenic hydrocarbons [16,17,18]. However, algae exhibit heterogeneous forms of lipids owing to their habitat variety and their changing compositions in response to changes in cultivation parameters, light intensities, pH values, temperatures, and nutrients [19]. To achieve this aim, high-throughput methods are preferred to quickly identify changes in lipid composition for both biofuel and nutraceutical uses [20,21,22].

A fast lipid assessment can be accomplished using matrix-assisted laser desorption ionization (MALDI) time-of-flight (ToF) MS because of its well-recognized advantages including high throughput, easy sample preparation, minimal sample requirements, and ability to handle salty samples. Using MALDI coupled with a ToF analyzer offers significant benefits, including high sensitivity, rapid acquisition, and an increased dynamic range for profiling over a wide range of molecular weights and negligible fragmentation of the molecules being analyzed, which is crucial for accurate identification. Traditional methods for analyzing these fatty molecules in algae have faced some limitations, such as the occurrence of matrix-related background or in-source decay effects by commonly used matrices like 2,5-dihydroxybenzoic acid (DHB) and α-cyano-4-hydroxycinnamic acid (CHCA), hindering proper identification. Significant progress has been made in the analysis of low-molecular-weight (LMW) molecules such as lipids, and other matrices [23,24] with better detection performances have been proposed. For instance, working in the negative ion mode provides simplified spectra and improves sensitivity for several analytes including fatty acids (FAs), lipids, and glycans as deprotonated molecules [25,26]. Some new promising basic matrices, such as 1,5-diaminonaphthalene (DAN) [27], 9-aminoacridine (9AA) [28], arylamines [29], and 1,8-bis(dimethylamino)naphthalene (DMAN), have recently been reported as being able to ionize labile compounds in the negative mode. Because of their well-recognized advantages for recovering reduced fragmentations, spot-to-spot reproducibility, and higher sensitivity at minor laser fluences, the use of binary matrices for MALDI analysis has also been proposed as a reliable choice [30,31,32,33,34].

Among them, 9AA and DAN matrices exhibit specific good features that can be exploited for algal lipidome investigation. While 9AA has been largely employed as a matrix for anionic PLs, sulfatides (STs), and cardiolipins (CLs), with few matrix-related ions [35], DAN has been applied as an electron-transfer matrix for the analysis of intact chlorophylls, avoiding demetallation and fragmentation of the phytol–ester linkage [36,37]. This study aims to showcase the effectiveness of a new binary matrix in analyzing lipid classes in algae samples and to highlight the advantages of using it over a single matrix. We successfully investigated an equimolar mixture of 9AA/DAN matrix to analyze both large algae species (i.e., Wakame, Dulse, Nori) and microscopic algae (namely, Spirulina, a cyanobacteria blue-green algae, and Chlorella, a single-celled green algae). This method significantly improved the detection of the different types of lipids present in the algae, allowing for a more comprehensive analysis of their lipid profiling.

## 2. Results and Discussion

### 2.1. Suitability of Selected Matrices for Lipid Analysis

A crucial factor to consider when evaluating new MALDI matrices is the minimization of unwanted fragment artefacts generated during the ionization process within the source. These artefacts, known as in-source decay (ISD) or in-source fragmentation (ISF) [38], are primarily influenced by the analyte’s chemical structure and the ionization conditions [39]. These conditions include factors like the ionization temperature, voltage, and laser fluence, which are all directly related to the chosen matrix [40]. While significant ISD can be valuable for gleaning the structural information of pure compounds using MALDI MS, minimal ISD is preferred for detailed analysis of lipid mixtures. This is because various fragmentation pathways can result in (i) isobaric lipid species, (ii) the interference of product ions with endogenous species, (iii) inaccurate quantitative comparisons, as the extent of fragmentation can vary between different lipid species within a class, hindering accurate quantification [41]. For example, imaging lysophospholipids (LPL, phospholipids lacking one fatty acyl chain, FAC) can be compromised when using a matrix with strong ISD effects, such as 2,5-DHB. This is because in-source losses of acyl chains from intact phospholipids can occur [42]. Previous research suggests that 9AA offers several advantages, including minimal background noise (a high interfering ion at just *m*/*z* 193.077), a high sensitivity, homogeneous matrix crystallization, and minimal ISD fragmentation [43]. Conversely, findings regarding 1,5-DAN are more diverse. It has been used as a matrix for both positive and negative mode lipid analysis [44], for chlorophylls without causing demetallation (viz., removal of Mg^2+^) [37], and even for cleaving disulfide bonds in peptides [45].

**Figure 1:**
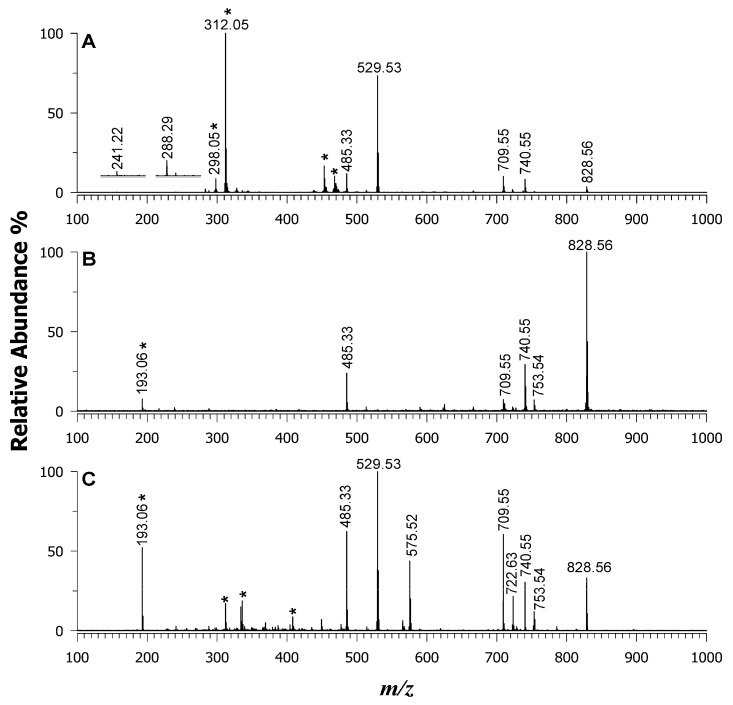
MALDI-MS of an EquiSPLASH™ LIPIDOMIX^®^ standard mixture of 13 deuterated standard lipids containing 15:0 and 18:1(d_7_) fatty acyl chains using DAN (**A**), 9AA (**B**), and the binary mixture of DAN/9AA (**C**) as matrices. The matrix signals are denoted by an asterisk.

In the current study, using a mixture of 13 deuterated standard lipids containing 15:0 and 18:1(d_7_) fatty acyl chains (see Section 3.1), the effects of the matrix such as DAN (plot A), 9AA (plot B), and their binary blend (plot C) on the ISD in the MALDI MS negative ion mode (Figure 1) were examined. As said, the ISD on the lipids could generate lyso-forms and signals arising from the loss of a FAC as a carboxylate ion and the polar head groups from deprotonated PLs ([M-H]^–^). Therefore, in the spectra, we searched for the presence of possible lyso-PLs, carboxylate anions at *m*/*z* 241.22 (FA 15:0) and 288.29 (FA 18:1(d_7_)), and/or the occurrence of lipids being hydrolyzed in the polar head. As a result, there was a lack of fragments corresponding to fatty acyl carboxylate anions for all the species investigated when using 9AA (plot B) and the binary matrix (plot C), while barely discernible peak signals were observed using the DAN matrix at *m*/*z* 241.22 and 288.29 (see zoom in Figure 1A). No peak signals generated from the loss of FA as ketenes or as fatty acids were observed in any of the MALDI MS spectra. A well-known polar head loss of serine is usually observed in phosphoserine (PS) species. In the standard mixture under investigation, the neutral loss of serine moiety from a deprotonated PS at *m*/*z* 753.54 should yield a fragment at *m*/*z* 666.52, which was absent in any spectrum, thus allowing us to conclude that the ISD effect was irrelevant for 9AA and the binary matrix and very low for DAN as a single matrix (Figure 1A). It is worthwhile to mention that the absence of fragments is likely due to the relatively low laser energy employed, which reduces both ISD and matrix interferences. An explanation of these findings could be related to good crystallization behaviour since a very homogenous spot was obtained using both single and binary matrices.

Another key factor to consider in testing novel matrices or pioneering mixtures is the ion suppression effect often observed in MALDI MS analyses of mixtures containing multiple analytes with different ionization efficiencies/proton affinities (PAs)/charge states [46]. Here, all the species were singly charged; therefore, the charge effect could be overlooked. Both matrices had highly similar PAs, working well in the negative ion mode, being 233 and 225 kcal/mol for 9AA and DAN, respectively [37]. Therefore, under these circumstances, the effects of PA were more analyte-dependent. For instance, acidic PLs such as phosphatidylinositols (PIs) are only detected in the negative ion mode, having a low PA. Meanwhile, sphingomyelins (SMs) and phosphatidylcholine (PCs) show relatively high PAs (260.4 and 255.3 kcal/mol [47], respectively) and are usually detected in the positive ion mode unless they generate an intact or demethylated radical ion, such as [M]^—•^ and [M-CH_3_]^—•^, respectively, using an electron transfer matrix. Phosphatidylglycerols (PGs) and phosphatidylethanolamines (PEs) with variable substituents (e.g., plasmalogen) were recorded in both ion modes due to their intermediate acidity and associated PAs. Looking at the standard mixture of deuterated lipids, the suppression effect of PIs in the negative ion mode is impressive when using 9AA, being the highest ion in the spectrum, while it is lowered in the DAN and binary mixture. Moreover, the basic PLs such as PCs, LPCs, and SMs were not detected using the 9AA, while they were barely seen, as radical anions, when DAN was employed, due to the electron transfer behavior of this matrix. In comparison, the binary matrix allowed us to obtain the highest species coverage in the number of identified lipids with comparable intensities (see relative intensity % in Table 1). In the case of ceramide, a formate adduct was observed as [M-H+HCOOH]^—^ at *m*/*z* 575.52, likely due to some formic acid impurities on the target or solvent. As expected, tri-(TG) and diacylglycerols (DG) neutral lipids were not uncovered in the negative ion mode. All the other detected species with their *m*/*z* values are summarized in Table 1.

### 2.2. Analysis of Wakame Lipid Extract

**Figure 2:**
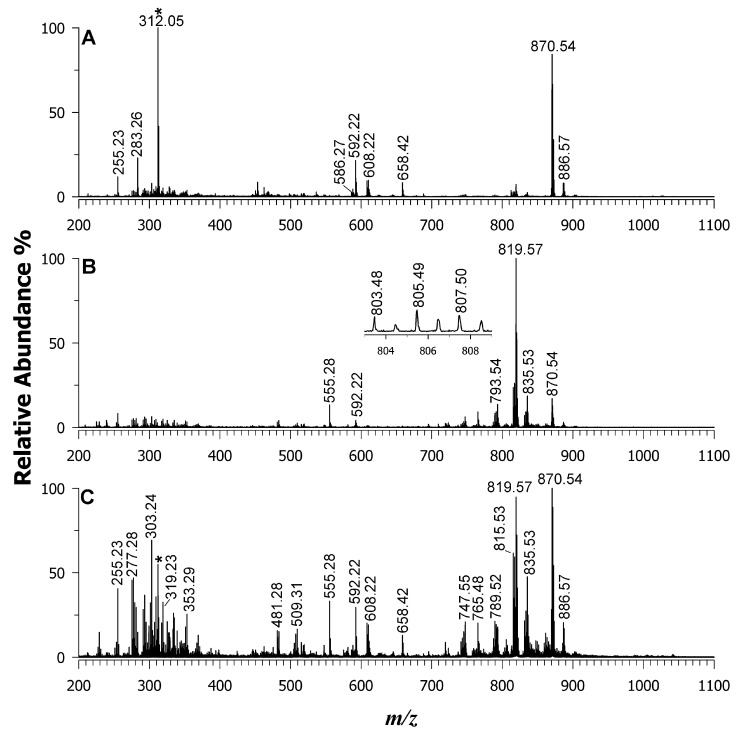
MALDI-MS of Wakame lipid extract using DAN (plot **A**), 9AA (plot **B**), and a binary DAN/9AA mixture (plot **C**) as matrices. The matrix signals are denoted by an asterisk.

To evaluate the binary matrix’s effectiveness in desorbing and ionizing lipids from food samples using MALDI MS, a representative algal extract from Wakame was analyzed in the negative ion mode. As described in the Experimental section, the lipid extract was deposited onto the target plate using the dried droplet deposition approach, which consistently produced a thin and homogeneous layer, as observed using a digital microscope. Figure 2 displays the MALDI mass spectra obtained from the sample extract using three different matrices: DAN (plot A), 9AA (plot B), and the binary mixture (plot C). Lipid assignments were facilitated by referencing the LIPID MAPS [48] and Alex^123^ [49] databases, as well as by performing tandem MALDI MS (MS/MS) analyses. Additionally, published data on the algal extract obtained using liquid chromatography coupled with MS [12] and existing literature [50] proved to be valuable resources for identifying the MALDI MS peak signals.

**Figure 3:**
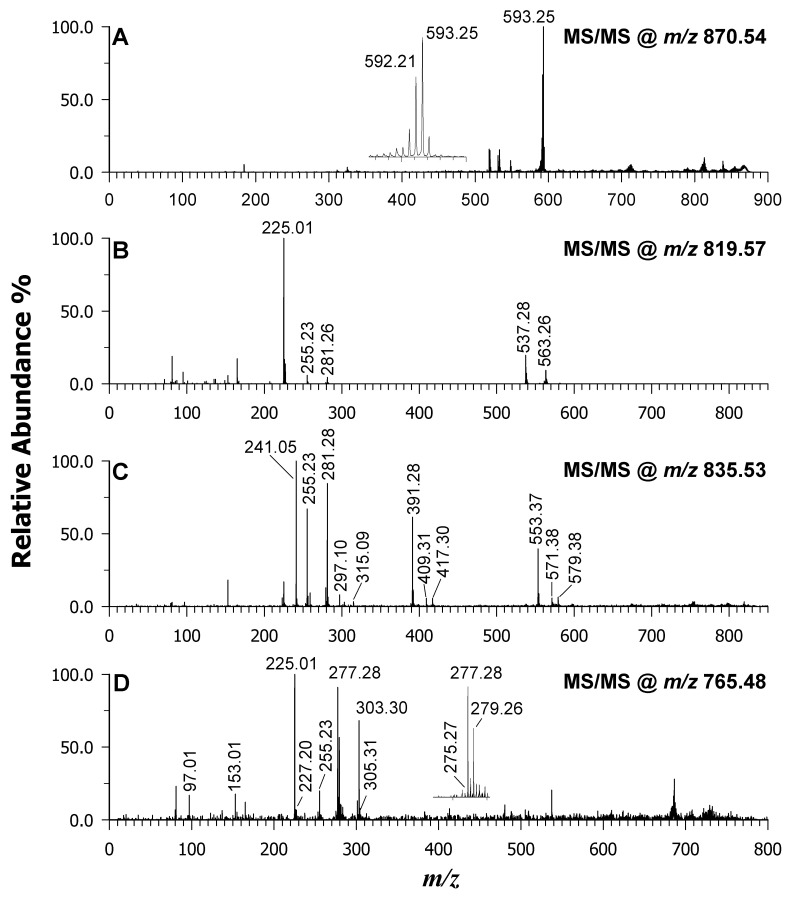
MALDI-MS/MS of *Pheophytin a* at *m*/*z* 870.54 (plot **A**), sulfoquinovosyldiacylglycerol at *m*/*z* 819.57 (plot **B**), phosphatidylinositol at *m*/*z* 835.53 (plot **C**), and sulfoquinovosyldiacylglycerol/phosphatidylglycerol at *m*/*z* 765.48 (plot **D**). DAN/9AA was used as a matrix.

An analysis of the algal lipidome revealed that the investigated *m*/*z* range encompassed ions that were consistent with several types of molecules. These included free (F)FAs as carboxylate anions (*m*/*z* 200–350), LPLs and lyso-GLs (*m*/*z* 300–550), PLs, GLs, chlorophylls (*m*/*z* 600–900), and arsenosugar phospholipids (*m*/*z* 900–1100). Significant variations in the relative intensities and the presence of specific species depending on the matrix used (DAN, 9AA, or binary mixture) were identified. While the peak signal at *m*/*z* 312.05 was due to the DAN matrix itself, the most intense peak at *m*/*z* 870.54 corresponded to [Chl *a* − Mg + 2H]^–•^. It is important to note that the DAN matrix was reported to detect intact chlorophyll *a*. However, the chlorophyll *a* of dried, stored, and thermally processed algae undergoes a yellow-brown colour change, which involves the natural release of magnesium ions and the conversion of chlorophylls *a* and *b* into their respective pheophytins (Pheo *a* and *b*) [51]. The confirmation was achieved using tandem MS on the precursor ion at *m*/*z* 870.54 (see Figure 3A), which after the neutral loss of the phytyl moiety and hydrogen migration generated two product ions at *m*/*z* 592.21 and *m*/*z* 593.25.

Figure 2A shows the mass spectrum obtained using the DAN matrix. Here, peak signals at *m*/*z* 586.27 and 658.42 were identified as phycocyanobilin and fucoxanthin pigments, respectively. However, a significant drawback of the DAN matrix is that these pigment signals can significantly suppress the ionization of lipid molecules. A contrasting picture emerges when using the 9AA matrix (Figure 2B). The most intense peak corresponded to a deprotonated molecule at *m*/*z* 819.57, tentatively assigned to SQDG 34:1. This species possesses 34 carbon atoms across both acyl chains with one unsaturation. Further confirmation of the lipid class comes from the MS/MS spectrum of this precursor ion (Figure 3B). A peak signal at *m*/*z* 225.01 was observed, corresponding to the formation of a dehydrated sulfoquinovosyl anion in the gas phase. Additionally, less-intense carboxylate anions were detected at *m*/*z* 255.23 (16:0) and 281.26 (18:1) as product ions. These FACs were further corroborated by two product ions arising from their neutral loss as FAs: *m*/*z* 537.28 (oleic acid loss) and *m*/*z* 563.26 (palmitic acid loss). Other SQDG species were revealed by signals at *m*/*z* 789.52, 791.53, 793.54, 815.498, and 817.514 [12]. Additionally, lyso-forms were observed at *m*/*z* 555.28, 579.26, and 581.31. As expected, the presence of a sulfonic group in the structure of the sulfoquinovosyl monoglycerides (SQMGs) and SQDGs makes them easily detectable as deprotonated species ([M-H]^–^) by using 9AA as a MALDI matrix. However, the signals of PIs were still observed at *m*/*z* 803.48, 805.49, 807.50 (see inset in Figure 2), 831.50, 833.52, and 835.53. Figure 3C showcases the MS/MS spectrum of the anion at *m*/*z* 835.53 as an example. An analysis of the signals at *m*/*z* 241.05, 297.10, and 315.09 suggested the PI class. This inference was further supported by three peak signals at *m*/*z* 391.28, 409.31, and 417.30, which corresponded to the neutral loss of a dehydrated inositol molecule (162.05 Da) from product ions detected at *m*/*z* 553.37, 571.38, and 579.38, respectively. Additionally, the product ions at *m*/*z* 255.23 and 281.28 suggested the presence of fatty acyl chains 16:0 and 18:1, tentatively assigning the molecule as PI 16:0_18:1. While other less-acidic species like PGs were also detected, isobaric SQDG species exhibited suppressed signals. A case study was reported in the MS/MS spectrum of the precursor ion at *m*/*z* 765.48 of Figure 3D, which could be identified as either SQDG 30:0 or PG 36:6 or even a combination of both due to their very close masses. The peak signal at *m*/*z* 225.01 suggested the presence of SQDG, in agreement with the peak signals at *m*/*z* 227.20 and 255.23 due to the 14:0 and 16:0 acyl chains of the SQDG 14:0_16:0. However, the presence of fragments at *m*/*z* 153.01 [glycerophosphate–H_2_O]^—^ and *m*/*z* 97.01 as [H_2_PO_4_]^—^ common to PAs and PGs and their lyso-forms hinted at the concomitant detection of an isobaric PL. Taking into account the precursor ion, along with the carboxylate anion at *m*/*z* 277.28, one possible assignment is PG 18:3/18:3. The occurrence of other fatty acyl chains at *m*/*z* 303.30, 305.31, 279.26, and 275.27 suggests the likely presence of isomeric PG 36:6 as PG 20:4_16:2, PG 20:5_16:1, or PG 18:4_18:2. The absence of a prior separation step resulted in the assignment of multiple identities to lipid species (see Table 2). When 9AA was employed as the matrix, the peak signals for pheophytin *a* along with its corresponding neutral phytyl loss fragment and several free fatty acids within the *m*/*z* range of 200–320 were observed, although with a lower intensity compared to DAN. This observation suggests that the 9AA matrix facilitates the deprotonation of a wider range of lipid species in the same algal extract compared to DAN.

**Figure 4:**
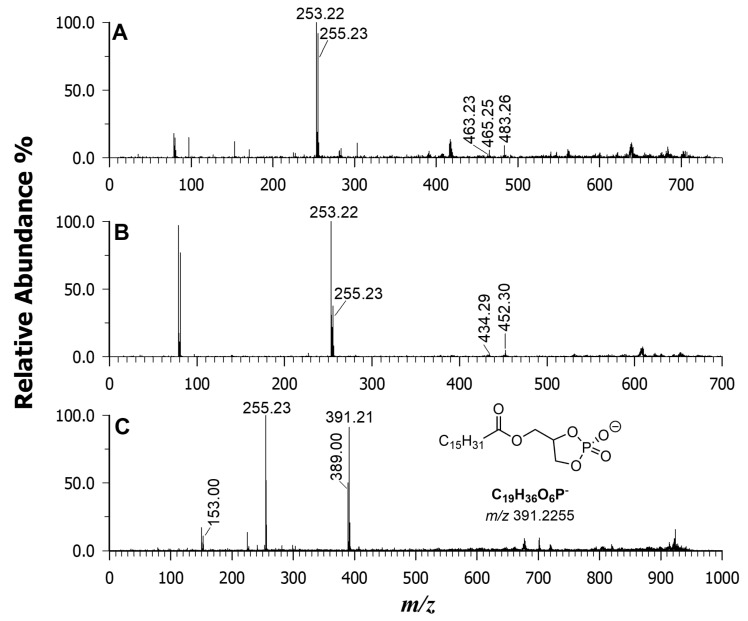
MALDI-MS/MS of phosphatidylglycerol at *m*/*z* 719.49 (plot **A**), phosphatidylethanolamine at *m*/*z* 688.50 (plot **B**), and arsenosugar PL at *m*/*z* 957.50 (plot **C**). Binary DAN/9AA was used as a matrix.

The combination of both matrices was tested, and the resulting MALDI MS spectrum is shown in Figure 2C. The spectrum displays a significant increase in the number of signals, and the relative intensities are more comparable between different classes. This indicates a substantial reduction in the suppression effect. Carefully looking at the *m*/*z* values between 200 and 1000, the intensities of the less-acidic PLs such as PGs and PEs were significantly enhanced. Plots A and B of Figure 4 display two representative MS/MS spectra of a PG at *m*/*z* 719.49 and a PE at *m*/*z* 688.50, respectively. The lipid calculator database of the precursor ion at *m*/*z* 719.49 suggested a PG (32:1), and the generation of two carboxylate anions of FA 16:0 (palmitate ion) and 16:1 (palmitoleate ion) in the gas phase at *m*/*z* 255.23 and 253.22, respectively, indicating a PG 16:0_16:1. Concerning plot B, which is the tandem MS spectrum of a PE, the two most intense product ions were again at *m*/*z* 255.23 and 253.22. As a result, the PE species was identified as 16:0_16:1. It is interesting to note that the most abundant FAs recovered in the fatty acyl chains corresponded to C16:0 and C16:1.

Remarkably, by using a binary matrix, very low-intensity peak signals were also recognized as lipid species. As an example, the relatively low content of arsenosugarphospholipids (As-PLs) in the Wakame sample extracts was distinguished and explained by the MALDI MS/MS analysis. The tandem MS spectrum of the precursor ion at *m*/*z* 957.50 (Figure 4C) exhibited the product ion at *m*/*z* 389.00 [C_10_H_19_AsO_9_P]^−^, which served as a diagnostic marker for the As-containing polar head. The single product ion at *m*/*z* 255.23 corresponded to the palmitate anion, together with the “d” ion at *m*/*z* 391.21 [C_19_H_36_O_6_P]^—^ (see inset in Figure 4C, where one of the possible structures is reported), suggested the As-PL was 16:0/16:0.

The same approach was applied to all the lipids detected in the seaweed samples. All of the putative assignments are summarized in Table 2, also highlighting where each species was found in the DAN, 9AA, and the DAN/9AA matrices. Using the equimolar binary matrix, a more informative fingerprint with a good signal-to-noise ratio was obtained, which was recognized as including FFAs, pigments, PIs, PEs, PGs, and much less abundant As-PLs.

### 2.3. Analysis of Micro and Macro Algae Extracts

**Figure 5:**
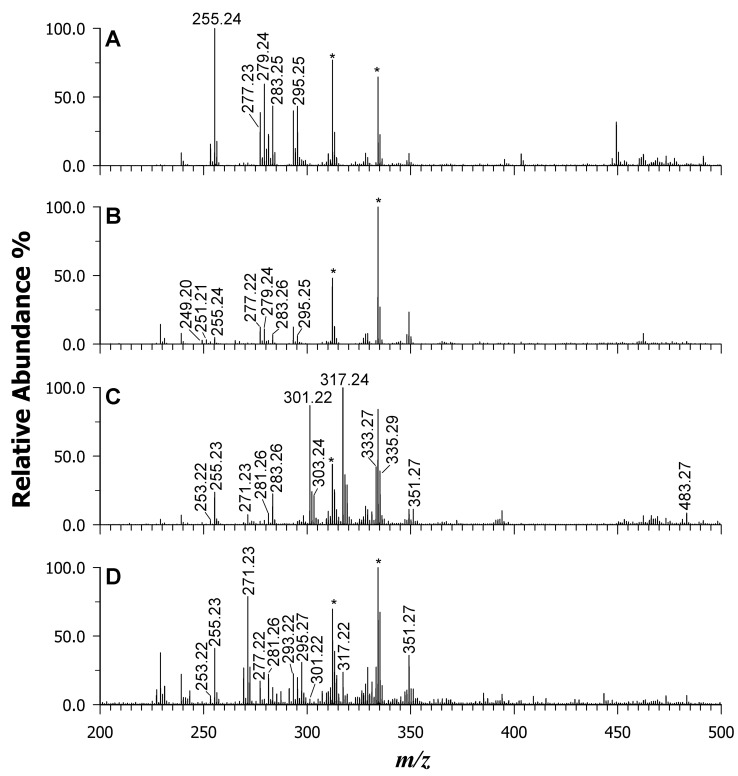
MALDI-MS in the *m*/*z* range of 200–500 of microalgae Spirulina (plot **A**), Chlorella (plot **B**), and macroalgae Dulse (plot **C**), and Nori (plot **D**) using the binary mixture of DAN/9AA. Matrix signals are indicated by *.

**Figure 6:**
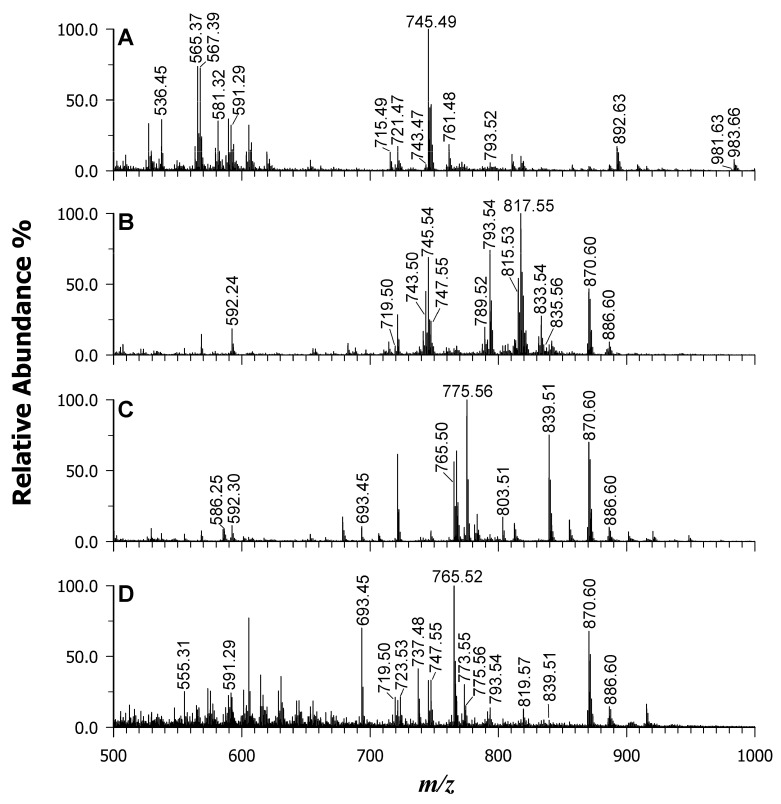
MALDI-MS in the *m*/*z* range of 500–1000 of microalgae Spirulina (plot **A**), Chlorella (plot **B**), macroalgae Dulse (plot **C**), and Nori (plot **D**) using the binary mixture of DAN/9AA.

To demonstrate the broader applicability of the binary DAN/9AA matrix to complex samples, lipid mass profiles were collected from a variety of micro- and macro-algae species. These included Spirulina and Chlorella in powdered form, as well as Dulse and Nori in dried-leaf form. For easier visual examination and comparisons, the results are presented in two figures: Figure 5 displays the low mass range (*m*/*z* 200–500), while Figure 6 focuses on the middle-high mass range (*m*/*z* 500–1000). The rationale behind this approach lies in the established variability in the fatty acid contents and compositions of algae. Several factors, including geographical location, water temperature and salinity, pH, growth stage, and environmental stress responses, have been shown to influence these characteristics. Consequently, a rapid and straightforward method for obtaining mass spectral “lipid fingerprints” becomes highly desirable.

Macroalgae are classified into three major groups: brown algae (*Phaeophyceae*), green algae (*Chlorophyta*), and red algae (*Rhodophyta*). Red and brown seaweeds are generally recognized for their high content of PUFAs, including linoleic acid (18:2), arachidonic acid (20:4), and sometimes eicosapentaenoic acid (20:5) as the major components. Conversely, blue-green seaweed such as Spirulina and green seaweed such as Chlorella tend to be dominated by saturated fatty acids such as palmitic acid (16:0) and monounsaturated fatty acids like palmitoleic acid (16:1) [52,53]. Figure 5 displays the MALDI mass spectra obtained for Spirulina (A), Chlorella (B), Dulse (C), and Nori (D) using the binary DAN/9AA matrix.

Each peak identified in the spectra corresponds to FFAs, LPLs, phospholipids (PLs), and pigments. Tables S1–S4 (Supplementary Materials) provide the peak lists and lipid assignments for all the examined algal extracts, further confirming the matrix’s remarkable potential for acquiring LMW compounds with minimal matrix interference (note that to recognize and label matrix peaks, a spot with just the matrix is always acquired as a control).

A comparison of all four samples revealed differences in the relative intensities of FAs. For example, the Chlorella sample displayed peak signals at *m*/*z* 249.20 and 247.21, corresponding to FAs 16:3 and 16:4, which are characteristic of chloroplast PLs. Conversely, red algae (i.e., Dulse and Nori) exhibited a higher intensity for FA 20:5 and 20:4, as previously reported by Schmid et al. [11] and Schlotterbeck et al. [50]. Figure 6 highlights even more interesting differences at the higher mass range. The existence of numerous PLs and GLs was confirmed (see data for Tables S1–S4), with slight variations in intensity. Additionally, the occurrence of other species with varying total carbon atoms and unsaturation levels likely reflects the distinct FA profiles. For instance, Spirulina (A) and Chlorella (B) microalgae displayed PG 34:4 (*m*/*z* 741.47), PG 34:3 (*m*/*z* 743.50), PG 34:2 (*m*/*z* 745.54), and PG 38:6 (*m*/*z* 793.54, isobaric with SQDG 32:0). In contrast, Nori (C) and Dulse (D) extracts preferentially revealed SQDG 36:5 (*m*/*z* 839.51), PG 36:1 (*m*/*z* 775.56), and PA 36:5 (*m*/*z* 693.45). Intriguingly, intact chlorophylls *a* and *b* were detected at *m*/*z* 892.63 and 908.55, respectively, only in Spirulina and Chlorella extracts that were not thermically treated. This finding suggests that the binary matrix does not exert the release of Mg^2+^ ions during the desorption/ionization process. Conversely, their absence in the Dulse, Nori, and Wakame samples is a possible consequence of the drying processes employed. Other pigments were also observed in the Spirulina sample: deprotonated diatoxanthin at *m*/*z* 565.34 ([C_40_H_54_O_2_-H]^—^ with monoisotopic mass *m*/*z* 565.4051), deprotonated zeaxanthin or zeaxanthol at *m*/*z* 567.35 ([C_40_H_56_O_2_-H]^—^ with monoisotopic mass *m*/*z* 567.4208), and lycopene radical anion at *m*/*z* 536.45 ([C_40_H_56_]^–•^ with monoisotopic mass *m*/*z* 536.4388) [54]. Additionally, di-galactosyl-diglycerides (DGDGs) were detected as formate adducts in the Spirulina specimen at *m*/*z* 981.63 (DGDG 36:6), 983.66 (DGDG 36:5), 999.61 (DGDG 36:5;1), 1005. 67 (DGDG 37:1), and 1007.69 (DGDG 37:0).

## 3. Materials and Methods

### 3.1. Chemicals

Water, acetonitrile, methanol, 1,5-diamino naphthalene (DAN), 9-aminoacridine (9AA), EquiSPLASH^®^ LIPIDOMIX^®^ solution containing 13 deuterated lipid standards at a concentration of 100 µg/mL each (PC 15:0/18:1(d7), LPC 18:1(d7), PE 15:0/18:1(d7), LPE 18:1(d7), PG 15:0/18:1(d7), PI 15:0/18:1(d7), PS 15:0/18:1(d7), TG 15:0/18:1(d7)/15:0, DG 15:0/18:1(d7), MG 18:1(d7), CE 18:1(d7), SM 18:1/18:1(d9), and ceramide 15(d7)) were obtained from Merck (Milan, Italy). All the solvents used were LC-MS grade except for CHCl_3_, which was HPLC grade. A mass standards kit for calibration was purchased from AB Sciex (Concord, ON, Canada). Edible algae (*Undaria pinnatifida* or Wakame, *Palmaria palmata* or Dulse, *Porphyra* spp. or Nori) and microalgae (*Arthrospira platensis* or Spirulina and *Chlorella vulgaris* or Chlorella) were bought from local markets. The labels indicated that the Dulse was collected on the coast of French Brittany, the Wakame was collected on the coast of Galicia, the Nori was harvested in the Pacific Northwest, and the Spirulina and Chlorella were grown in ecologically protected basins in China.

### 3.2. Instrumentation

All the experiments were performed using a 5800 MALDI ToF/ToF analyzer (AB SCIEX, Darmstadt, Germany) equipped with a neodymium-doped yttrium lithium fluoride (Nd:YLF) laser (345 nm) in the reflectron negative mode with a typical mass accuracy of 5 ppm. At least 6000 laser shots were typically accumulated in the MS mode using a random rastering pattern with a laser pulse rate of 400 Hz, whereas in the MS/MS mode spectra, up to 8000 laser shots were acquired and averaged using a pulse rate of 1000 Hz. The MS/MS experiments were performed by setting a potential difference between the source and the collision cell of 1 kV. Ambient air was used as the collision gas, with a medium pressure of 10^−6^ Torr. The delayed extraction (DE) time was set to 240 ns.

DataExplorer software 4.0 (AB Sciex) was used to control the acquisitions and to perform the initial elaboration of data, while SigmaPlot 14.0 was used to graph the final mass spectra. ChemDraw Pro 8.0.3 (CambridgeSoft Corporation, Cambridge, MA, USA) was employed to draw the chemical structures.

### 3.3. Sample Preparation

#### 3.3.1. Extraction of Lipids

Lipid extraction was performed by following a Bligh and Dyer (BD) protocol [55]. Briefly, a sample of dried seaweed (about 0.2 g) was placed in a test tube, soaked in water, and left for 20 min to rehydrate it and remove excess salt. The water was thrown away, and the wet sample was placed in a clean test tube along with 3 mL of a solution of CH_3_OH/CHCl_3_ 2:1 (*v*/*v*) and 800 µL of water. The system was vortexed and stored at room temperature for one hour. Afterwards, 1 mL of CHCl_3_ and 1 mL of H_2_O were added to the sample. The system was vortexed once again and centrifuged for 10 min at 3000× *g*. The chloroform phase was recovered, dried under an N_2_ flow, dissolved in 100 μL of CH_3_OH/CHCl_3_ (1:1, *v*/*v*), and analyzed using MALDI MS. For the microalgae, such as spirulina and chlorella, the protocol was the same, starting from 100 mg of weighed product.

#### 3.3.2. MALDI MS Analysis

For the MALDI analysis, 5 µL of lipid extract were mixed (1:1, *v*/*v* ratio) with each matrix prepared at a 0.030 M concentration in the appropriate solvent (acetone or methanol). DAN and 9AA stock solutions (0.06 M) were prepared in MeOH or acetone. The binary DAN/9AA matrix was obtained by properly mixing the above stock solutions. After testing various matrix ratios (90/10, 30/70, 50/50, 70/30, 10/90, *v*/*v*) and two solvents, we observed that the best conditions in terms of the number of peaks and S/N ratio were obtained using a DAN/9AA equimolar mixture in acetone. A total of 1 µL of the analyte matrix mixture was spotted directly on the target plate, dried in the dark, and analyzed using MALDI MS. Unless otherwise specified, the dried-droplet method was thoroughly used in this work. After drying, the spots were observed using a Ninyoon 4K WiFi 50–1000× Digital Microscope (Shenzhen, Futianqu, China) which allowed the observation of uniform crystals within the spot.

## 4. Conclusions

A new MALDI binary matrix composed of 1,5-DAN and 9AA was applied for the lipidome characterization of micro- and macroalgae. After testing the low ISD and lowered analyte suppression effects on a standard lipid mixture, a fast lipidomic signature was easily achieved on complex samples since deprotonated molecules were invariably formed alongside the radical anions for pigments. Free fatty acids, lyso- and phospholipids, lyso- and glycolipids, and pigments were detected, highlighting the most significant differences among the five algal samples. The MALDI-MS/MS spectra confirmed the lipid identification in a high-throughput array format. The use of a binary matrix creates uniform sample spots, which allows for the use of lower laser energy settings and the generation of assorted libraries of lipid species. In account of such advantages, many samples can be quickly analyzed without needing to purify each lipid beforehand.

## Figures and Tables

**Table 1:** List of standard lipids detected by employing DAN, 9AA, and binary mixture DAN/9AA as matrices for MALDI MS. The relative intensity % is indicated for each species compared to the base peak.

Lipid Species	[M-H]^–^ *m*/*z*	DAN	9AA	DAN/9AA (Equimolar)
FA (15:0)	241.22	0.9		
FA (18:1(d7))	288.29	1.8		
PC 15:0_18:1(d7)	752.60 [M]^–•^			3.3
LPC 18:1(d7)	528.38 [M]^–•^			5.6
PE 15:0_18:1(d7)	709.55	11	6.2	61
LPE 18:1(d7)	485.33	12	24	63
PG 15:0_18:1(d7)	740.55	8.5	30	31
PI 15:0_18:1(d7)	828.56	3.9	100	33
PS 15:0_18:1(d7)	753.54		4.4	12
SM 18:1_18:1(d9)	722.63 [M-CH_3_]^–•^	2.7		22
C15 ceramide-d7	529.53/	74		100
	575.52 [M-H+HCOOH]^—^			44

**Table 2:** List of all lipid species in the Wakame algal extract identified using MALDI MS using DAN, 9AA, and a binary DAN/9AA mixture as matrices.

Observed *m*/*z*	Theoretical *m*/*z*	Error (ppm)	Suggested Identification	Matrix		
				DAN	9AA	Binary
199.16	199.170	−50	[FA 11:0–H]^—^			X
213.18	213.186	−28	[FA 12:0–H]^—^	X		X
225.18	225.186	−27	[FA 14:1–H]^—^		X	X
227.20	227.202	−9	[FA 14:0–H]^—^	X		X
251.21	251.202	32	[FA 16:2–H]^—^		X	X
253.22	253.217	12	[FA 16:1–H]^—^	X	X	X
255.23	255.233	−12	[FA 16:0–H]^—^	X	X	X
271.23	271.228	7	[FA 16:0;1–H]^—^	X	X	X
275.21	275.202	29	[FA 18:4–H]^—^	X	X	X
277.22	277.217	11	[FA 18:3–H]^—^	X	X	X
279.24	279.233	25	[FA 18:2–H]^—^	X	X	X
281.26	281.249	39	[FA 18:1–H]^—^	X	X	X
283.26	283.248	42	[FA 18:0–H]^—^	X	X	X
291.22	291.233	−45	[FA 18:4;1–H]^—^	X	X	X
293.22	293.212	27	[FA 18:3;1–H]^—^	X	X	X
295.27	295.263	24	[FA 18:2;1–H]^—^	X	X	X
297.25	297.244	20	[FA 18:1;1–H]^—^	X	X	X
299.25	299.259	−30	[FA 18:0;1–H]^—^			X
301.22	301.217	10	[FA 20:5–H]^—^			X
303.24	303.233	23	[FA 20:4–H]^—^	X	X	X
307.26	307.264	−13	[FA 20:2–H]^—^	X	X	X
309.27	309.280	−32	[FA 20:1–H]^—^	X	X	X
311.28	311.295	−48	[FA 20:0–H]^—^			X
317.22	317.212	25	[FA 20:5;1–H]^—^	X	X	X
319.23	319.228	6	[FA 20:4;1–H]^—^	X	X	X
325.26	325.275	−46	[FA 20:1;1–H]^—^	X	X	X
327.23	327.233	−9	[FA 22:6–H]^—^			X
333.27	333.280	−30	[FA 22:3–H]^—^	X	X	X
335.29	335.296	−18	[FA 22:2–H]^—^	X	X	X
339.33	339.326	12	[FA 22:0–H]^—^			X
343.21	343.228	−52	[FA 22:6;1–H]^—^			X
351.27	351.290	−57	[FA 22:2;1–H]^—^	X	X	X
353.29	353.306	−45	[FA 22:1;1–H]^—^	X	X	X
369.32	369.337	−46	[FA 23:0;1–H]^—^			X
381.32	381.337	−45	[FA 24:1;1–H]^—^			X
424.27	424.247	54	[LPE 14:0–H]^—^			X
450.27	450.263	16	[LPE 16:1–H]^—^	X		X
453.24	453.225	33	[LPG 14:1–H]^—^	X		
479.27	479.242	58	[LPG 16:2–H]^—^			X
481.28	481.257	48	[LPG 16:1–H]^—^	X	X	X
483.27	483.273	−6	[LPG 16:0–H]^—^		X	X
505.28	505.257	46	[LPG 18:3–H]^—^			X
507.29	507.273	34	[LPG 18:2–H]^—^	X	X	X
509.31	509.288	43	[LPG 18:1–H]^—^	X	X	X
515.23	515.226	8	[LPI 12:0–H]^—^	X	X	X
528.33	528.310	38	[LPC 20:4–CH_3_]^—^			X
536.43	536.438	−15	[Lycopene]^–•^	X		
555.28	555.284	−7	[SQMG 16:0–H]^—^		X	X
579.26	579.284	−41	[SQMG 18:2–H]^—^		X	X
581.31	581.300	17	[SQMG 18:1–H]^—^		X	X
586.27	586.280	−17	[Phycocyanobilin diacid]^–•^ [Phycoerythrobilin]^–•^	X		X
590.28	590.310	−51	[Phycourobilin]^–•^	X		X
591.29	591.261	49	[Pheo a-phytil–H]^–•^	X		X
592.22	592.269	-83	[Pheo a-phytil]^–•^	X		X
608.22	608.263	−71	[Pheo b-phytil]^–•^	X		X
658.42	658.424	−6	Fucoxanthin	X		X
688.50	688.492	12	[PE 32:1–H]^—^			X
695.50	695.466	49	[PA 36:4–H]^—^		X	
709.39	709.420	−42	[SQDG 26:0–H]^—^		X	X
717.51	717.471	54	[PG 32:2–H]^—^		X	X
719.49	719.487	4	[PG 32:1–H]^—^		X	X
	719.466	33	[PA 38:6–H]^—^			
723.53	723.497	46	[PA 38:4–H]^—^		X	X
737.48	737.439	56	[PG 36:4–H]^—^		X	X
741.48	741.450	40	[PA 40:9–H]^—^			X
743.50	743.487	17	[PG 34:3–H]^—^		X	X
	743.466	46	[PA 40:8–H]^—^			
745.54	745.503	50	[PG 34:2–H]^—^	X	X	X
747.55	747.518	43	[PG 34:1–H]^—^	X	X	X
765.48	765.483	−4	[SQDG 30:0–H]^—^		X	X
	765.471	12	[PG 36:6–H]^—^			
773.55	773.534	21	[PG 36:2–H]^—^		X	
775.56	775.549	14	[PG 36:1–H]^—^		X	
787.50	787.467	42	[SQDG 32:3–H]^—^		X	X
789.52	789.483	47	[SQDG 32:2–H]^—^		X	X
791.53	791.498	40	[SQDG 32:1–H]^—^		X	X
793.54	793.503	47	[PG 38:6–H]^—^		X	X
	793.514	33	[SQDG 32:0–H]^—^			
801.52	801.456	80	[PI 32:4–H]^—^		X	X
	801.565	−56	[PG 38:2–H]^—^			
803.51	803.472	47	[PI 32:3–H]^—^		X	X
	803.581	−88	[PG 38:1–H]^—^			
805.49	805.487	4	[PI 32:2–H]^—^		X	X
807.50	807.503	−4	[PI 32:1–H]^—^		X	X
813.52	813.483	45	[SQDG 34:4–H]^—^	X	X	X
815.53	815.498	39	[SQDG 34:3–H]^—^	X	X	X
817.55	817.514	44	[SQDG 34:2–H]^—^	X	X	X
819.57	819.530	49	[SQDG 34:1–H]^—^	X	X	X
829.52	829.487	40	[PI 34:4–H]^—^		X	X
	829.514	7	[SQDG 35:3–H]^—^			
831.50	831.503	−4	[PI 34:3–H]^—^	X	X	X
833.52	833.519	1	[PI 34:2–H]^—^	X	X	X
835.53	835.534	−5	[PI 34:1–H]^—^	X	X	X
839.53	839.472	69	[PI 35:6–H]^—^		X	X
	839.498	38	[SQDG 36:5–H]^—^			
841.55	841.487	75	[PI 35:5–H]^—^		X	X
	841.514	43	[SQDG 36:4–H]^—^			
843.56	843.503	68	[PI 35:4–H]^—^		X	X
	843.530	36	[SQDG 36:3–H]^—^			
845.54	845.519	25	[PI 35:3–H]^—^			X
	845.545	−6	[SQDG 36:2–H]^—^			
847.53	847.534	−5	[PI 35:2–H]^—^		X	X
	847.561	−37	[SQDG 36:1–H]^—^			
849.54	849.550	−12	[PI 35:1–H]^—^		X	X
	849.577	−44	[SQDG 36:0–H]^—^			
851.55	851.566	−19	[PI 35:0–H]^—^			X
861.52	861.483	43	[SQDG 38:8–H]^—^	X	X	X
863.53	863.566	−42	[PI 36:1–H]^—^		X	X
	863.498	37	[SQDG 38:7–H]^—^			
865.54	865.581	−47	[PI 36:0–H]^—^		X	X
	865.514	30	[SQDG 38:6–H]^—^			
867.55	867.530	23	[SQDG 38:5–H]^—^		X	X
869.56	869.545	17	[SQDG 38:4–H]^—^		X	X
870.54	870.566	−30	[Pheo a]^–•^	X		X
885.59	885.550	45	[PI 38:4–H]^—^	X	X	X
886.57	886.561	10	[Pheo b]^–•^	X		X
957.50	957.506	−6	[As-PL 32:0–H]^—^			X

Legend: FA = fatty acid; LPE = lysophosphatidylethanolamine; LPG = lysophosphatidylglycerol; LPI = lysophosphatidylinositol; LPC = lysophosphatidylcholine; SQMG = sulfoquinovosylmonoacylglycerol; SQDG = sulfoquinovosyldiacylglycerol; PG = phosphatidylglycerol; PA = phosphatidic acid; PI = phosphatidylinositol; PE = phosphatidylethanolamine; As-PL = diacylarsenosugar phospholipids; Pheo = pheophytin.

## Data Availability

The data are available from the corresponding author upon request.

## Supplementary Materials

The supporting information can be downloaded at: https://www.mdpi.com/article/10.3390/ijms25115919/s1.
