# Supplementary material for: Development of a New Binary Matrix for the Comprehensive Analysis of Lipids and Pigments in Micro- and Macroalgae Using MALDI-ToF/ToF Mass Spectrometry
Source: Int J Mol Sci. 2024 May 29;25(11):5919. doi: 10.3390/ijms25115919 (PMC11172705; doi:10.3390/ijms25115919)
Supplement: Supplementary file 1 [file ijms-25-05919-s001.zip › ijms-3014863-supplementary.pdf]

Supplementary information

Development of a new binary matrix for the comprehensive analysis of lipids and pigments in micro- and macroalgae by MALDI-ToF/ToF mass spectrometry

Mariachiara Bianco [1], Giovanni Ventura [1,2], Davide Coniglio [1], Antonio Monopoli [1], Ilario Losito [1,2], Tommaso R.I. Cataldi [1,2] and Cosima D. Calvano *[1,2]

[1]Dipartimento di Chimica, [2]Centro di Ricerca Interdipartimentale S.M.A.R.T. Università degli Studi di Bari "Aldo Moro", Campus Universitario, Via E. Orabona, 4 - 70126 Bari, Italy

*Correspondence: Author for correspondence, email: *cosimadamiana.calvano@uniba.it*

Keywords: MALDI MS/MS; chlorophylls; lipids; alga; binary matrix

Table S1. List of all lipid species in the Spirulina microalgal extract identified by MALDI MS using DAN/9AA as a matrix.

Observed *m/z*	Theoretical *m/z*	Error (ppm)	Suggested identification
239.21	239.201	38	[FA 15:1 – H]$^{-}$
253.21	253.217	-28	[FA 16:1 – H]$^{-}$
255.24	255.233	27	[FA 16:0 – H]$^{-}$
277.23	277.217	47	[FA 18:3 – H]$^{-}$
279.24	279.233	25	[FA 18:2 – H]$^{-}$
281.25	281.249	4	[FA 18:1 – H]$^{-}$
283.25	283.248	7	[FA 18:0 – H]$^{-}$
293.22	293.212	27	[FA 18:3;1 – H]$^{-}$
295.25	295.263	-44	[FA 18:2;1 – H]$^{-}$
297.24	297.244	-13	[FA 18:1;1 – H]$^{-}$
299.25	299.259	-30	[FA 18:0;1 – H]$^{-}$
395.23	395.220	25	[LPA 15:0 – H]$^{-}$
449.25	449.267	-38	[LPA 19:1 – H]$^{-}$
	449.231	42	[LPA 18:2;1 – H]$^{-}$
453.24	453.225	33	[LPG 14:1 – H]$^{-}$
507.28	507.273	14	[LPG 18:2 – H]$^{-}$
509.30	509.288	24	[LPG 18:1 – H]$^{-}$
527.25	527.253	-6	[SQMG 14:0 – H]$^{-}$
536.45	536.438	22	[Lycopene]$^{-\bullet}$
563.32	563.335	-27	[LPG 22:2 – H]$^{-}$
565.37	565.351	34	[LPG 22:1 – H]$^{-}$
567.39	567.367	41	[LPG 22:0 – H]$^{-}$
579.27	579.284	-24	[SQMG 18:2 – H]$^{-}$
581.32	581.300	34	[SQMG 18:1 – H]$^{-}$
583.30	583.316	-27	[SQMG 18:0 – H]$^{-}$
591.29	591.261	49	[Pheo a- phytil – H]$^{-\bullet}$
593.27	593.273	-5	[LPI 18:3 – H]$^{-}$
595.26	595.289	-49	[LPI 18:2 – H]$^{-}$
603.26	603.284	-40	[SQMG 20:4 – H]$^{-}$
605.33	605.300	50	[SQMG 20:3 – H]$^{-}$
607.29	607.316	-43	[SQMG 20:2 – H]$^{-}$
619.31	619.288	36	[LPI 20:4 – H]$^{-}$
621.33	621.305	40	[LPI 20:3 – H]$^{-}$
623.29	623.320	-48	[LPI 20:2 – H]$^{-}$
715.49	715.456	48	[PG 32:3 – H]$^{-}$
717.47	717.471	-1	[PG 32:2 – H]$^{-}$
719.47	719.487	-24	[PG 32:1 – H]$^{-}$
	719.466	6	[PA 38:6 – H]$^{-}$
721.47	721.503	-46	[PG 32:0 – H]$^{-}$
723.51	723.497	18	[PA 38:4 – H]$^{-}$
743.47	743.487	-23	[PG 34:3 – H]$^{-}$
	743.466	5	[PA 40:8 – H]$^{-}$
745.49	745.503	-17	[PG 34:2 – H]$^{-}$
747.50	747.518	-24	[PG 34:1 – H]$^{-}$
761.48	761.439	54	[PG 36:8 – H]$^{-}$
765.48	765.483	-4	[SQDG 30:0 – H]$^{-}$
	765.471	12	[PG 36:6 – H]$^{-}$
767.52	767.487	43	[PG 36:5 – H]$^{-}$
769.50	769.503	-4	[PG 36:4 – H]$^{-}$
771.53	771.518	16	[PG 36:3 – H]$^{-}$

773.56	773.534	34	[PG 36:2 – H]$^{-}$
775.51	775.549	-50	[PG 36:1 – H]$^{-}$
787.45	787.467	-22	[SQDG 32:3 – H]$^{-}$
789.50	789.483	22	[SQDG 32:2 – H]$^{-}$
791.46	791.498	-48	[SQDG 32:1 – H]$^{-}$
793.52	793.503	21	[PG 38:6 – H]$^{-}$
	793.514	8	[SQDG 32:0 – H]—
815.48	815.498	-22	[SQDG 34:3 – H]$^{-}$
817.52	817.514	7	[SQDG 34:2 – H]$^{-}$
819.55	819.530	24	[SQDG 34:1 – H]$^{-}$
831.49	831.503	-16	[PI 34:3 – H]$^{-}$
833.49	833.519	-35	[PI 34:2 – H]$^{-}$
835.52	835.534	-17	[PI 34:1 – H]$^{-}$
837.57	837.549	25	[PI 34:0 – H]$^{-}$
870.58	870.566	16	[Pheo a] $^{-\bullet}$
886.52	886.561	-46	[Pheo b]$^{-\bullet}$
892.63	892.644	-16	[NAPE 46:4 – H]$^{-}$
894.61	894.659	-55	[NAPE 46:3 – H]$^{-}$
981.63	981.652	-30	[DGDG 39:4 – H]$^{-}$
983.66	983.668	-8	[DGDG 39:3 – H]$^{-}$
957.53	957.506	25	[As-PL 32:0-H]$^{-}$

Legend: FA=fatty acid; LPA=lysophoshatidic acid; LPE=lysophosphatidylethanolamine; LPG=lysophosphatidylglycerol; LPI=lysophosphatidylinositol; LPC=lysophosphatidylcholine; SQMG=sulfoquinovosylmonoacylglycerol; SQDG=sulfoquinovosyldiacylglycerol; DGDG=digalactosyldiglyceride; PG=phosphatidylglycerol; PA= phosphatidic acid; PI=phosphatidylinositol; PE=phosphatidylethanolamine; NAPE=N-acyl-phosphatidylethanolamine As-PL=diacylarsenosugar phospholipids; Pheo=pheophytin .

Table S2. List of all lipid species in the Chlorella microalgal extract identified by MALDI MS using DAN/9AA as matrix.

Observed *m/z*	Theoretical *m/z*	Error (ppm)	Suggested identification
239.21	239.201	38	[FA 15:1 – H]⁻
249.20	249.186	56	[FA 16:3 – H]⁻
251.21	251.202	32	[FA 16:2 – H]⁻
253.21	253.217	-28	[FA 16:1 – H]⁻
255.24	255.233	27	[FA 16:0 – H]⁻
277.22	277.217	11	[FA 18:3 – H]⁻
279.24	279.233	25	[FA 18:2 – H]⁻
281.25	281.249	4	[FA 18:1 – H]⁻
283.26	283.248	42	[FA 18:0 – H]⁻
291.23	291.233	-10	[FA 18:4;1 – H]⁻
293.20	293.212	-41	[FA 18:3;1 – H]⁻
295.25	295.263	-44	[FA 18:2;1 – H]⁻
297.27	297.244	42	[FA 18:1;1 – H]⁻
349.28	349.274	17	[FA 22:3;1 – H]⁻
481.26	481.257	6	[LPG 16:1 – H]⁻
483.26	483.273	-27	[LPG 16:0 – H]⁻
505.24	505.257	-34	[LPG 18:3 – H]⁻
507.28	507.273	14	[LPG 18:2 – H]⁻
555.26	555.284	-43	[SQMG 16:0 – H]⁻
592.24	592.269	-49	[Pheo a- phytil]⁻•
688.49	688.492	-3	[PE 32:1–H]⁻
710.50	710.477	32	[PE 34:4–H]⁻
712.49	712.492	-3	[PE 34:3–H]⁻
714.52	714.508	17	[PE 34:2–H]⁻
719.50	719.487	18	[PG 32:1 – H]⁻
	719.466	47	[PA 38:6 – H]⁻
721.53	721.503	37	[PG 32:0 – H]⁻
741.47	741.450	27	[PA 40:9 – H]⁻
743.50	743.487	17	[PG 34:3 – H]⁻
	743.466	46	[PA 40:8 – H]⁻
745.54	745.503	50	[PG 34:2 – H]⁻
747.55	747.518	43	[PG 34:1 – H]⁻
765.48	765.483	-4	[SQDG 30:0 – H]⁻
	765.471	-14	[PG 36:6 – H]⁻
767.47	767.487	-22	[PG 36:5 – H]⁻
769.50	769.503	-4	[PG 36:4 – H]⁻
787.49	787.467	29	[SQDG 32:3 – H]⁻
789.52	789.483	47	[SQDG 32:2 – H]⁻
791.52	791.498	28	[SQDG 32:1 – H]⁻
793.54	793.503	47	[PG 38:6 – H]⁻
	793.514	33	[SQDG 32:0 – H]⁻
795.51	795.518	-10	[PG 38:5 – H]⁻
803.53	803.472	72	[PI 32:3 – H]⁻
	803.581	-76	[PG 38:1 – H]⁻
805.47	805.487	-21	[PI 32:2 – H]⁻
807.48	807.503	-28	[PI 32:1 – H]⁻
815.53	815.498	39	[SQDG 34:3 – H]⁻
817.55	817.514	44	[SQDG 34:2 – H]⁻
819.56	819.530	37	[SQDG 34:1 – H]⁻
821.54	821.545	-6	[SQDG 34:0 – H]⁻

831.50	831.503	-4	[PI 34:3 – H]$^{-}$
833.54	833.519	25	[PI 34:2 – H]$^{-}$
835.56	835.534	31	[PI 34:1 – H]$^{-}$
839.48	839.472	10	[PI 35:6 – H]$^{-}$
	839.498	-21	[SQDG 36:5 – H]$^{-}$
841.50	841.487	15	[PI 35:5 – H]$^{-}$
	841.514	-17	[SQDG 36:4 – H]$^{-}$
843.51	843.503	8	[PI 35:4 – H]$^{-}$
	843.530	-24	[SQDG 36:3 – H]$^{-}$
870.60	870.566	39	[Pheo a]$^{-\bullet}$
886.60	886.561	44	[Pheo b]$^{-\bullet}$

Table S3. List of all lipid species in the Dulse macroalgal extract identified by MALDI MS using DAN/9AA as matrix.

Observed *m/z*	Theoretica *m/z*	Error (ppm)	Suggested identification
239.20	239.201	-4	[FA 15:1 – H]$^{-}$
249.18	249.186	-24	[FA 16:3 – H]$^{-}$
253.22	253.217	12	[FA 16:1 – H]$^{-}$
255.23	255.233	-12	[FA 16:0 – H]$^{-}$
271.23	271.228	7	[FA 16:0;1 – H]$^{-}$
277.22	277.217	11	[FA 18:3 – H]$^{-}$
279.22	279.233	-47	[FA 18:2 – H]$^{-}$
281.26	281.249	39	[FA 18:1 – H]$^{-}$
283.26	283.248	42	[FA 18:0 – H]$^{-}$
301.22	301.217	10	[FA 20:5 – H]$^{-}$
303.24	303.233	23	[FA 20:4 – H]$^{-}$
305.24	305.249	-29	[FA 20:3 – H]$^{-}$
317.24	317.248	-25	[FA 21:4 – H]$^{-}$
319.25	319.264	-44	[FA 21:3 – H]$^{-}$
327.22	327.233	-40	[FA 22:6 – H]$^{-}$
329.24	329.249	-27	[FA 22:5 – H]$^{-}$
331.25	331.264	-42	[FA 22:4 – H]$^{-}$
333.27	333.280	-30	[FA 22:3 – H]$^{-}$
335.29	335.296	-18	[FA 22:2 – H]$^{-}$
347.26	347.259	3	[FA 22:4;1 – H]$^{-}$
349.27	349.274	-11	[FA 22:3;1 – H]$^{-}$
351.27	351.290	-57	[FA 22:2;1 – H]$^{-}$
453.24	453.225	33	[LPG 14:1 – H]$^{-}$
455.23	455.242	-26	[LPG 14:0 – H]$^{-}$
481.27	481.257	27	[LPG 16:1 – H]$^{-}$
483.27	483.273	-6	[LPG 16:0 – H]$^{-}$
529.26	529.257	6	[LPG 20:5 – H]$^{-}$
537.33	537.319	20	[LPG 20:1 – H]$^{-}$
555.27	555.284	-25	[SQMG 16:0 – H]$^{-}$
586.25	586.280	-51	[Phycocyanobilin diacid]$^{-\bullet}$
			[Phycoerythrobilin]$^{-\bullet}$
592.30	592.269	52	[Pheo a- phytil] $^{-\bullet}$
601.25	601.269	-32	[SQMG 20:5 – H]$^{-}$
603.30	603.284	27	[SQMG 20:4 – H]$^{-}$
605.27	605.300	-50	[SQMG 20:3 – H]$^{-}$
607.29	607.316	-43	[SQMG 20:2 – H]$^{-}$
608.24	608.263	-38	[Pheo b- phytil]$^{-\bullet}$
693.45	693.450	0	[PA 36:5 – H]$^{-}$
721.51	721.503	10	[PG 32:0 – H]$^{-}$
723.51	723.497	18	[PA 38:4 – H]$^{-}$
737.46	737.439	28	[PG 36:4 – H]$^{-}$
747.54	747.518	29	[PG 34:1 – H]$^{-}$
765.50	765.483	22	[SQDG 30:0 – H]$^{-}$
	765.471	38	[PG 36:6 – H]$^{-}$
767.48	767.487	-9	[PG 36:5 – H]$^{-}$
769.50	769.503	-4	[PG 36:4 – H]$^{-}$
773.55	773.534	21	[PG 36:2 – H]$^{-}$
775.56	775.549	14	[PG 36:1 – H]$^{-}$
777.57	777.565	6	[PG 36:0 – H]$^{-}$
781.48	781.502	-28	[PG 37:5 – H]$^{-}$

783.52	783.518	3	[PG 37:4 – H]$^{-}$
785.53	785.534	-5	[PG 37:3 – H]$^{-}$
789.49	789.483	9	[SQDG 32:2 – H]$^{-}$
791.52	791.498	28	[SQDG 32:1 – H]$^{-}$
793.53	793.503	34	[PG 38:6 – H]$^{-}$
	793.514	20	[SQDG 32:0 – H]$^{-}$
803.51	803.472	47	[PI 32:3 – H]$^{-}$
805.48	805.487	-9	[PI 32:2 – H]$^{-}$
839.51	839.472	45	[PI 35:6 – H]$^{-}$
	839.498	14	[SQDG 36:5 – H]$^{-}$
841.49	841.487	4	[PI 35:5 – H]$^{-}$
	841.514	-29	[SQDG 36:4 – H]$^{-}$
843.52	843.503	20	[PI 35:4 – H]$^{-}$
	843.530	-12	[SQDG 36:3 – H]$^{-}$
855.52	855.503	20	[PI 36:5 – H]$^{-}$
857.52	857.519	1	[PI 36:4 – H]$^{-}$
870.60	870.566	39	[Pheo a]$^{-\bullet}$
885.58	885.550	34	[PI 38:4 – H]$^{-}$
886.60	886.561	44	[Pheo b]$^{-\bullet}$
901.56	901.581	-23	[PI 39:3 – H]$^{-}$

Table S4. List of all lipid species in the Nori macroalgal extract identified by MALDI MS using DAN/9AA as matrix.

Observed *m/z*	Theoretical *m/z*	Error (ppm)	Suggested identification
239.19	239.201	-46	[FA 15:1 – H]$^{-}$
243.20	243.197	12	[FA 14:0;1 – H]$^{-}$
253.22	253.217	12	[FA 16:1 – H]$^{-}$
255.23	255.233	-12	[FA 16:0 – H]$^{-}$
271.23	271.228	7	[FA 16:0;1 – H]$^{-}$
277.22	277.217	11	[FA 18:3 – H]$^{-}$
279.23	279.233	-11	[FA 18:2 – H]$^{-}$
281.26	281.249	39	[FA 18:1 – H]$^{-}$
283.25	283.248	7	[FA 18:0 – H]$^{-}$
291.23	291.233	-10	[FA 18:4;1 – H]$^{-}$
293.22	293.212	27	[FA 18:3;1 – H]$^{-}$
295.27	295.263	24	[FA 18:2;1 – H]$^{-}$
297.23	297.244	-47	[FA 18:1;1 – H]$^{-}$
299.26	299.259	3	[FA 18:0;1 – H]$^{-}$
301.22	301.217	10	[FA 20:5 – H]$^{-}$
307.25	307.264	-46	[FA 20:2 – H]$^{-}$
317.22	317.212	25	[FA 20:5;1 – H]$^{-}$
319.24	319.228	38	[FA 20:4;1 – H]$^{-}$
329.24	329.249	-27	[FA 22:5 – H]$^{-}$
331.25	331.264	-42	[FA 22:4 – H]$^{-}$
333.28	333.280	0	[FA 22:3 – H]$^{-}$
335.29	335.296	-18	[FA 22:2 – H]$^{-}$
347.25	347.259	-26	[FA 22:4;1 – H]$^{-}$
349.29	349.274	46	[FA 22:3;1 – H]$^{-}$
351.27	351.290	-57	[FA 22:2;1 – H]$^{-}$
353.30	353.306	-17	[FA 22:1;1 – H]$^{-}$
385.32	385.311	23	[FA 26:5 – H]$^{-}$
387.31	387.327	-44	[FA 26:4 – H]$^{-}$
409.24	409.236	10	[LPA 16:0 – H]$^{-}$
429.21	429.205	12	[LPA 18:4 – H]$^{-}$
431.21	431.220	-23	[LPA 18:3 – H]$^{-}$
455.24	455.242	-4	[LPG 14:0 – H]$^{-}$
483.28	483.273	14	[LPG 16:0 – H]$^{-}$
505.27	505.257	26	[LPG 18:3 – H]$^{-}$
507.28	507.273	14	[LPG 18:2 – H]$^{-}$
509.30	509.288	24	[LPG 18:1 – H]$^{-}$
555.31	555.284	47	[SQMG 16:0 – H]$^{-}$
575.25	575.253	-5	[SQMG 18:4 – H]$^{-}$
577.25	577.269	-33	[SQMG 18:3 – H]$^{-}$
591.29	591.261	49	[Pheo a- phytil – H]$^{-\bullet}$
592.24	592.269	-49	[Pheo a- phytil]$^{-\bullet}$
601.28	601.269	18	[SQMG 20:5 – H]$^{-}$
603.29	603.284	10	[SQMG 20:4 – H]$^{-}$
605.29	605.300	-17	[SQMG 20:3 – H]$^{-}$
630.39	630.414	-38	[PE 28:2 – H]$^{-}$
693.45	693.450	0	[PA 36:5 – H]$^{-}$
719.50	719.487	18	[PG 32:1 – H]$^{-}$
	719.466	47	[PA 38:6 – H]$^{-}$
723.53	723.497	46	[PA 38:4 – H]$^{-}$
737.48	737.439	56	[PG 36:4 – H]$^{-}$

745.52	745.503	23	[PG 34:2 – H]⁻
747.55	747.518	43	[PG 34:1 – H]⁻
765.52	765.483	48	[SQDG 30:0 – H]⁻
	765.471	64	[PG 36:6 – H]⁻
773.55	773.534	21	[PG 36:2 – H]⁻
775.56	775.549	14	[PG 36:1 – H]⁻
791.52	791.498	28	[SQDG 32:1 – H]⁻
793.54	793.503	47	[PG 38:6 – H]⁻
	793.514	33	[SQDG 32:0 – H]—
803.52	803.472	35	[PI 32:3 – H]⁻
805.47	805.487	-21	[PI 32:2 – H]⁻
807.52	807.503	21	[PI 32:1 – H]⁻
815.52	815.498	27	[SQDG 34:3 – H]⁻
817.55	817.514	44	[SQDG 34:2 – H]⁻
819.57	819.530	49	[SQDG 34:1 – H]⁻
831.53	831.503	32	[PI 34:3 – H]⁻
833.54	833.519	25	[PI 34:2 – H]⁻
835.54	835.534	7	[PI 34:1 – H]⁻
839.51	839.472	45	[PI 35:6 – H]⁻
	839.498	14	[SQDG 36:5 – H]⁻
870.60	870.566	39	[Pheo a]⁻•
885.58	885.550	34	[PI 38:4 – H]⁻
886.60	886.561	44	[Pheo b]⁻•
915.59	915.597	-8	[PI 40:3 – H]⁻
